# Contributions to Reparative Surgery

**Published:** 1877-07

**Authors:** 


*Contributions to Reparative Surgery.* By Gurdon Buck, M. D. New York: D. Appleton & Co.

The decease of the author in March last followed not long after the publishing of this volume. He was appointed attending surgeon to the New York Hospital in 1837, and held this position until his death. Eminent in his personal character and ability, he held numerous positions of honor and trust; he was widely known for the boldness and skill of his operations, and the painstaking care he gave his cases. He is universally known for his establishment of the method of weight and pulley extension in fractures of the thigh. In this work plastic surgery, a matter of his especial pride is treated of. Numerous cases accompanied by faithful illustrations are carefully detailed, and his success in contractions from burns, cheiloplasty and hare lip is a matter of much interest.

